# Wireless Readout of Multiple SAW Temperature Sensors

**DOI:** 10.3390/s19143077

**Published:** 2019-07-12

**Authors:** Gudrun Bruckner, Jochen Bardong

**Affiliations:** Carinthian Tech Research (CTR AG), 9524 Villach, Austria

**Keywords:** SAW, sensors, wireless, delay lines, industrial application, 2.45 GHz

## Abstract

It has since long been known that surface acoustic wave (SAW) devices, resonators as well as delay lines, can be used as passive wireless sensors for physical quantities, like temperature and pressure, as well as gas sensors or identification-tags (ID-tags). The sensors are robust, work passively without a battery, can be applied at high temperatures, and provide a high resolution. Nevertheless, if the devices are used wirelessly in an industrial environment, several constraints have to be taken into account, especially when more than one quantity or device needs to be measured at the same time. The paper addresses the challenges that must be tackled when establishing multi-sensor-wireless-readout for industrial applications. Major issues here are the legal regulations for industrial, scientific and medical frequency bands (ISM-bands), as well as sampling time and costs, which impose severe restrictions to any system design. We describe several design approaches and their constraints. We successfully designed sensors based on reflective delay lines that allow the parallel readout of four independent temperature sensors in the 2.45 GHz ISM-band. These devices were fabricated and positively tested, demonstrating the applicability of SAW sensors for industrial applications.

## 1. Introduction to SAW Sensors

SAW (surface acoustic wave) sensors can provide significant advantages in industrial sensing as they can be readout wirelessly, work passively, and can sustain high temperatures. It has been shown by many groups that different physical and chemical quantities can be measured with these devices [1,2,3,4,5,6,7,8,9,10]. While the sensors themselves allow a very wide span of operation, like temperatures from close to −273 °C to 800 °C [3,11,12], working frequencies from 100 kHz to several GHz, and various quantities that can be measured, the field becomes significantly narrower if the restrictions given by ISM-band regulations and practical considerations, like antenna size, sampling frequency, and cost, have to be taken into account. If several sensors have to be readout at the same time, the limitations add up even further. This paper describes the general design considerations that have to be taken into account if SAW sensors are to be used in industrial applications and demonstrates a successful example for a wireless simultaneous readout of four temperature sensors.

The following introduction is intended for readers not familiar with the field of SAW sensors. It should help users to understand the features and constraints of these devices and assist in selecting the right technology for their application.

A general introduction to SAW devices can be found in [1,2,13,14,15]. Sensing with SAW devices is, for example, described in [1,16,17], resonators in [18,19,20], delay lines in [21,22,23], SAW based radio frequency identification tags (RFID-tags) in [2,24,25,26,27], and reader units in [28,29].

SAW sensors can be divided into two groups: (one port) resonators and (reflective) delay lines (RDL).

Resonators as resonant devices show narrow signals in the frequency domain, with peaks at their resonance frequency and their corresponding anti-resonance. The higher the quality factor, the narrower the peak(s). Effects like temperature or strain shift the maximum of these peaks in frequency, which can then be tracked in the reading device.

Measurement is done via the emission of an electromagnetic radio frequency (RF) wave by a reader unit. The wave is received by an antenna connected with the sensor and converted to an SAW on the piezoelectric substrate of the device by the interdigital transducer (IDT) structure. While the physical principles of wave generation and propagation are the same for resonators and delay lines, the latter are designed as devices with a wide transfer function that maximizes the bandwidth and thus shows very well defined sharp peaks of the impulse responses in the time domain. As the propagation time and frequency of the SAW are both influenced by the physical effect the device is exposed to, an analysis of the changes in either frequency (resonators) or delay time (RDL) provides the desired sensor information when the wave is retransmitted to the reader and analyzed there.

Table 1 gives a short comparison of the main resonator and delay line properties.

The change of the peak position in frequency or time due to temperature changes is described by the temperature coefficient of frequency (TCF) and delay (TCD), respectively. Generally, TCF = −TCD for a given material.

In the following discussion, temperature measurements and delay lines are used as examples, but equivalent considerations can be made for resonators and any measure (e.g., pressure, strain, mass loading).

The delay time *t*, at a given temperature *T*, is calculated relative to the delay time at the reference temperature *T*_0_, in a quadratic approximation as:

*T* = *t* (*T*_0_)·(1 + *TCD*_1_·(*T* − *T*_0_) + *TCD*_2_·(*T* − *T*_0_)^2^) (1)

For sensing applications, the linear temperature coefficient (*TCD*_1_) should be big to gain a large sensing effect and hence a high resolution. To avoid ambiguities when calculating the temperature from the measured signal shift, the corresponding shift in the time delay must be close to linear within the range of the sensor operation. For example, a second- or third-order characteristic with a turn over point within the temperature range would not allow the correct temperature to be resolved from the measured shift in the delay time. Hence, only substrate materials with monotonically increasing or decreasing temperature shifts can be applied for sensor devices. Usually a second order polynomial with a very small second order coefficient is desired for SAW sensors. While these considerations may sound trivial, they limit the choice of suitable sensor materials and applicable temperature ranges considerably. The applicability of piezoelectric substrates for SAW sensors can be described by the coupling coefficient (*k*^2^), a measure to describe the conversion efficiency from an electromagnetic radio frequency (RF) wave to a piezomechanical SAW and back, the propagation velocity of the SAW on the material surface, of the SAW’s amplitude attenuation while travelling over the surface of the crystal and the TCD, which, as a material property, describes the changes of the propagation velocity and the thermal expansion under temperature. For temperature sensors, of course, the stability of the material itself against decomposition and against the loss of the piezoelectric effect at high temperatures is an additional prerequisite.

All these properties are defined by the material constants and depend strongly on the chosen orientation in the crystal. For most materials, a trade-off between optimal temperature stability, high SAW velocity, and RF/SAW-coupling has to be found. For example, most substrates that are stable at very high temperatures (>800 °C), like lanthanum-gallium-silicate (Langasite, LGS), show small coupling coefficients (about 0.2%–0.4%) and low SAW velocities, while materials with coupling coefficients of several percent, like lithiumniobate (LN) or lithiumtantalate (LT), are limited in long term operation to temperatures of about ~ 300 °C [30].

Although it might be tempting to project a multi-purpose SAW sensor system, generally speaking, each application requires a dedicated sensor design. For example, the targeted environment (metal, dust, liquids, corrosives, electromagnetic shields) and the available space for the antenna have to be considered as well as the desired readout distance, data sampling rate, and operation frequency. The higher the operation frequency of the system, the smaller the corresponding antennas, but generally, the energy losses over the transmission path increase as well. For longer reading distances, antennas with higher gains and hence bigger dimensions have to be applied, which is often limited by practical considerations.

## 2. Multi Sensor Design

The aim of this work was to realize at least four independent SAW temperature sensors that can be readout simultaneously with a single reader antenna but without letting the four different sensor signals interfere with each other. The operation temperature should be up to 300 °C. The mutual temperature difference between the sensor locations can be as high as 250 °C. The minimum sampling rate of a readout of all four sensors was about 1 Hz. High temperature gradients are neither expected in space (along the sensor length) nor in time. As the sensors should be applied in free field measurements, ISM regulations must be observed.

The sensors were designed by careful consideration of the material properties and boundary conditions and selection of appropriate substrate and electrode materials. The different design steps are described in detail in the following section and lead to the choice of a reflective delay line design. The devices were fabricated and investigated experimentally.

A temperature reading was demonstrated by measuring S-parameters of the sensors with a vector network analyzer (VNA) at different positions within a tube furnace applying various temperature levels between room temperature and 270 °C. The data was analyzed with dedicated analysis scripts (Matworks Matrix Laboratory (MatLab^®^)) to retrieve the relevant sensor data, like the delay time, phase information at the peak and signal quality, and from them, the temperature reading for each sensor was deduced. The main steps of the data analysis were:

- Hanning window on S-parameter data.
- Inverse Fourier transform (IFFT) of the windowed S-parameters to the time domain, with applied zero padding to get 2048 data points for the 80 MHz bandwidth (2.4–2.48 GHz) and 8192 points for the 320 MHz bandwidth (2.29–2.61 GHz), respectively.
- Peak time and phase value detection of all sensor peaks.
- Assigning the peak data to the individual sensors.
- Computing the temperature from the measured delay time and phase value by applying the known TCD coefficients (polynomial of the second order) of lithiumniobate.

The performance in wireless reading was demonstrated by applying two different reading systems and setting up different experimental configurations by variation of the reading antennas, cables, and reading distance. The obtained data sets were analyzed in terms of signal strength and resolution versus distance of the antenna link.

### 2.1. Design Considerations

As resonator-based sensors impose fewer demands on the coupling coefficient of the substrate and allow the fabrication of small sensor elements, we at first considered a resonator based design. If four resonators have to be readout simultaneously, each one of them must work at a different frequency. This can be achieved by applying FDMA (frequency domain multiple access) designs as described, for example, in [31]. When using multiple resonators for temperature sensing purposes in the same antenna readout cone, the temperature-induced frequency shifts of sensors with adjacent center frequencies must not overlap at the maximum mutual temperature difference they are designed for, nor can any sensor’s center frequency be allowed to move out of the limits of the ISM-band.

A single resonator can be easily designed for the ISM-band at 433 MHz. This low operation frequency would allow the use of a well-established material, like quartz, or a high-temperature sustaining material, like LGS, as the piezoelectric SAW substrate. In addition, the low frequency means fewer losses in the free field propagation and on the substrate, and results in relatively wide electrode structures, which enhance the temperature stability of the sensor electrodes. However, a short calculation applying Equation (1) shows that the frequency shift due to temperature results in a shift of the devices’ center frequency by several MHz for 300 °C even for a low TCF of 30 ppm/°C. As the ISM-band at 433 MHz is only ~ 1.8 MHz wide, no feasible temperature sensor can be realized for the wide operation temperature of ~300 °C within that ISM-band, especially as two resonators are often applied to allow for differential measurements [16,17,18,32].

As the coefficient for pressure or strain is much smaller than the temperature effect, pressure and strain measurements can still be performed within the narrow band [32].

The only ISM-band available for wide band sensor applications is the ISM-band at 2.45 GHz that allows an operation between about 2400 and 2480 MHz. If four resonator sensors have to work within this bandwidth, the available frequency range must be divided in a meaningful manner, allowing the resonators to have different working temperatures and considering some safety margins. Hence, less than 20 MHz are available for the frequency shift of each sensor. One solution could be to reduce the temperature range of operation and/or to apply substrates with lower TCF. For example, a substrate with a TCF of 40 ppm/°C would allow for an operation range of 200 °C per resonator sensor. One should be aware that while selecting substrates with a lower TCF is possible, the sensitivity of the sensor will be reduced as well. Examples of commercially available substrates with low TCD besides quartz are LT 42° Y-cut with a TCD of ~ 40 ppm/°C or 36° Y-cut with a TCD of ~ 30 ppm/°C [33], which operate with SSBWs (surface skimming bulk waves). For resonator systems, the loss in sensitivity can be compensated by a sufficiently precise frequency measurement, but this may induce a longer sampling time and/or additional equipment costs on the reader side [19]. In addition, designing resonators with high Q-values is challenging for high frequencies. The combination of a low Q-factor and high frequency leads to a very short decay time constant of the resonator, meaning that reliable data can only be sampled within the short time interval after the pure electric reflections have been decayed and before the response of the resonators has vanished as well. While such solutions can be found for special applications, these are strong arguments against a resonator based design at 2.45 GHz.

For delay lines, as wide band devices, a high coupling coefficient, *k*^2^, is essential. Substrates with a high coupling, like LN and LT, can be applied to temperatures up to ~ 300 °C, which is sufficient for the targeted application. LN-YZ cut has a very high TCF of ~ –94 ppm/°C [34] and is hence ideal for temperature measurements.

**Figure 1:**
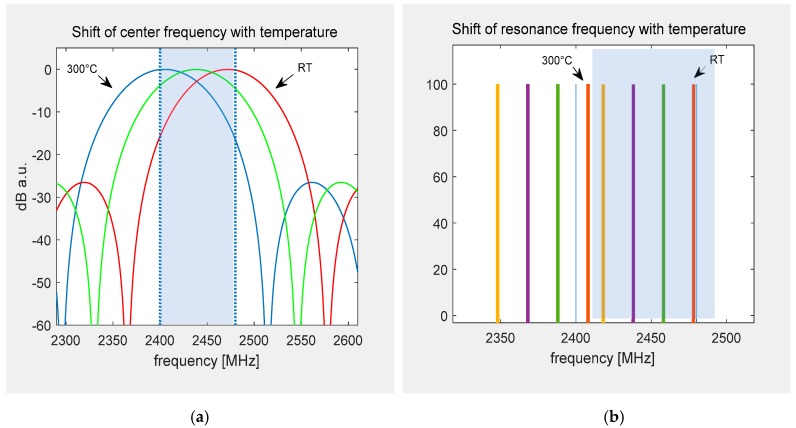
Sketch of the frequency shift due to an operation temperature of 300 °C with respect to the ISM-band for delay lines and resonators: (**a**) While only a part of the transfer function stays within the band at RT and 300 °C (red, blue), the devices can still be interrogated. The optimal operation point is set to 150 °C (green). (**b**) Using resonators, only one (red) stays in the band. The other devices marked as green, violet, and yellow get shifted out of the band when the temperature shift of ~280 °C is applied.

While the frequency shift due to temperature is the same for delay lines and resonators, the analysis in the time domain and the possible wide bandwidth of the sensor itself lead to a different behavior. The sketch in Figure 1 demonstrates the different situations when using resonators or delay lines. Both figures show a draft of the expected sensor response in the frequency domain in arbitrary units. For resonators, each sensor works at a different center frequency and if this frequency maximum shifts out of the band due to the temperature effect, the devices’ response cannot be evaluated any more. With delay lines, the sensors can be designed such that they have a wide identical bandwidth in the frequency domain so that their transfer function always overlaps with the dedicated ISM band no matter the temperature-induced frequency shift. Thus, an interrogation sweep will always lead to a successful readout. The example below (Figure 1a) shows a device with a center frequency of almost 2480 MHz at room temperature (RT) to allow the frequency to be shifted down by 66 MHz over the whole temperature range. The IDTs center frequency is designed such that the best performance is given in the middle of the temperature region (~150 °C), where most of the operation is expected. A simple analytical model that describes the IDT as a sin(x)/x function [14] was used for the plots of the delay lines (Figure 1a) whereas the frequencies of the four different resonators are simply indicated as lines (Figure 1b). In this example, the different resonators’ frequencies (red, green, violet, yellow) have the maximum available offset of 20 MHz. When a temperature shift from RT to 300 °C occurs, only one resonator (red) stays in the band, but is still interfering with three other devices. The other devices marked as green, violet, and yellow get shifted out of the band when the same temperature shift is applied.

Following this discussion, we decided to use a reflective delay line design with TDMA (time domain multiple access) for our sensors. TDMA means that all peaks must be well distinguishable in the time domain no matter how the different peaks are shifted by the temperature. In addition, the design must be suitable for phase tracking (see Section 2.2. below). Delay lines provide a wider design freedom for the time positioning of the sensor peaks compared to what resonators allow for in the frequency domain, as the maximum suitable time is only limited by the propagation losses on the delay path. Practically, the position of the latest peak should be kept below 4 µs for 2.45 GHz devices. We implemented a reflective delay line design because it halves the physical length of the sensor compared to a delay line with two IDTs. Furthermore, a reflective SAW delay line needs only one signal port that is connected to the antenna.

As described in the introduction, the temperature value of the sensor is derived from the shift of the impulse peaks in the time domain. If we are considering a differential delay time of about 1 µs, the shift per 0.15° is only ~14 ps. This resolution cannot be achieved by peak detection in the time domain, as the bandwidth is limited to ~80 MHz and hence the distance between the sampling points in the time domain is 12.5 ns. The resolution in peak detection can be enhanced, depending on the signal to noise ratio (SNR), by applying signal processing tools, like zero padding and parabolic peak fitting. To further increase the temperature readout accuracy, especially for weak signals, a phase reading algorithm must be applied to gain the desired resolution of 0.15 °C. Examples of different methods and applications of phase analysis can be found in [27,35,36,37,38].

### 2.2. Phase Analysis

While the phase value at the peak level can be detected within the precision of a few percent within 2π, additional information is required to determine the number of phase rotations and hence deduce the most accurate time-shift value possible. This information is provided by measuring the time-shift for reflective delay lines with different lengths. We studied designs with three and four peaks per sensor at different positions. One peak serves as a reference for differential measurements, the longest delay line is used for the actual temperature reading, and at least one additional shorter delay line is needed to resolve the phase ambiguity.

**Figure 2:**
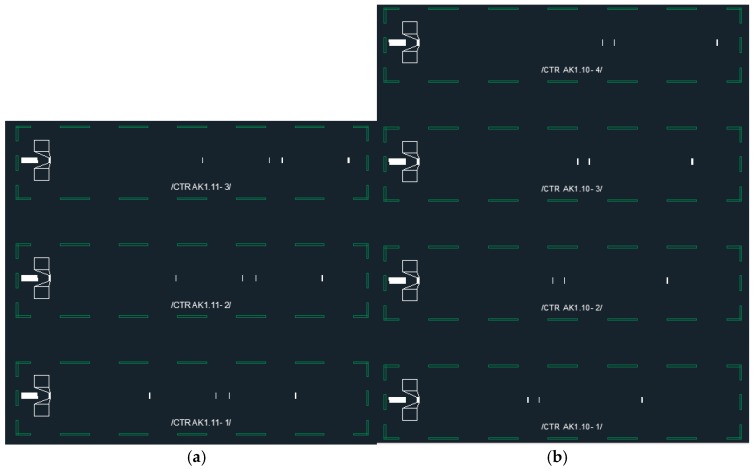
Mask design of two different multi sensor layouts based on reflective delay lines: The IDT structure where the SAW is generated can be seen to the very left of each device where the square wire bond pads are shown as well. The reflectors that correspond to the sensor peaks are visible as short white vertical lines. The white structure on the left of the IDT is a sink, which dampens the part of the wave emitted to the left side and thus reduces reflections from the edges of the die. (**a**) Design A: four peak design for three sensors. (**b**) Design B: three peak design for four different sensors.

Figure 2 shows two different designs (design A with four peaks and design B with three peaks) based on reflective delay lines that were fabricated. For both, an initial delay of at least 1 µs was used to separate the sensor response from pure electric crosstalk during the propagation path. The actual delay lines for the temperature reading are 1 µs and 1.4 µs long. The short delay lines have a length of 100 ns and 130 ns, respectively. After determining the time and phase values at each peak, the temperature can be calculated in a few steps: First, the time shift and the phase change of the long delay line are calculated. The phase information would allow for a very accurate temperature reading provided the number of phase rotations is known. The measurement of the long delay time is used to estimate this number of rotations, but with an uncertainty of a few periods, leading to a variance of the temperature estimation of better than 10 °C. To finally solve this ambiguity, the phase reading of the short delay line is applied. For the short delay line, the phase shift stays within one period of 2 π for 10 °C and hence the phase information of the short delay line can be used to identify the correct number of periods of the long delay line. Finally, the corrected number of periods and the phase information of the long delay line are combined to obtain the precise temperature information.

This method is only accurate if the temperature is the same for both delay lines. The placement of the short delay line in the middle of the long one allows operation with some spatial gradient as long as the mean temperature values of both delay lines are identical. Nevertheless, we finally opted for the second design (B) as we did not expect strong gradients in our application. As the latter design comprises only three peaks, it finally results in a shorter layout and therefore in lower losses. In addition, four sensors can be realized at a sensor length of 6 mm whereas only three sensors could be realized with the other design (see Figure 2).

In addition to the two designs described above, many more possible sensor concepts can be envisioned. One approach could be to simply use three peaks per sensor and merely arrange the sensors one after the other such that each reflector has its own and unique time position. On the other hand, this would result in very short delay lines and therefore in low resolution. Another possibility that was studied was to modify the first design with four reflectors in a way that one of the middle reflectors is omitted and the short delay line is not fabricated physically but is represented by the difference of the delay between first and second peak and second and third peak. As one reflector is removed, all the lengths can be reduced so that again, four sensors can be placed within the chip length of 6 mm. However, this design was very sensitive to temperature gradients along the chip length and was hence finally discarded, and we thus finally selected the two designs presented in Figure 2 for fabrication. The sensors have an area of 1.25 mm × 6 mm, and comprise a uniformly spaced IDT with a pitch of 0.7 µm and an aperture of 80 times of the wavelength. Thus, the center frequency at room temperature is located close to the upper limit of the ISM-band. The devices were processed on LN-YZ using an I-Line lithography system and evaporated aluminum as the electrode material and were mounted into a Kovar housing by Vectron International.

## 3. Results

### 3.1. Device Performance at Room Temperature

All fabricated devices (three- and four-peak designs) were measured on the wafer level and showed good performance. As expected, design (B) with three peaks per sensor has a slightly better SNR and a larger number of sensors and was hence selected for further investigations.

**Figure 3:**
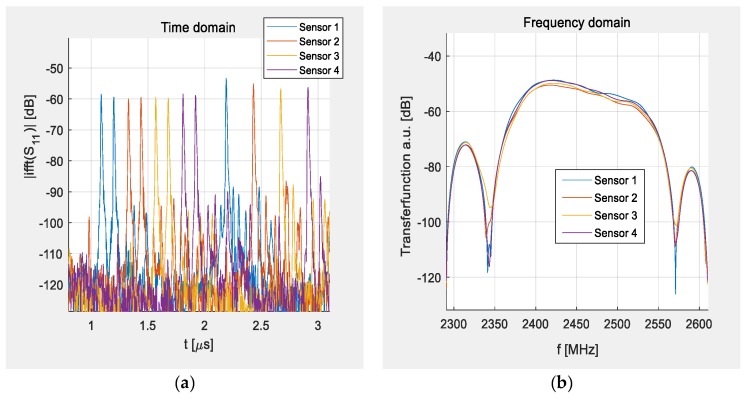
Measured S-parameters of four individual sensors: (**a**) left: time domain, the different sensor responses are shown in different colors; (**b**) right: frequency domain, all sensors show the same transfer function—a wideband plateau so that the interrogation in the ISM band can happen successfully no matter of the temperature shift.

Figure 3 shows the extracted time and frequency responses, measured separately for all four sensors. The time response shows the absolute value of the inverse Fourier transform (IFFT) of single port reflective S-parameter measurements after a Hanning window was applied. The frequency response was obtained from the time response by applying a Tukey window with a width of a few ns in the time domain around the signal of the first impulse peak (time-gating) and conducting a Fourier transformation back to the frequency domain. It can be seen that the frequency response (Figure 3b) is mostly identical for all sensors while the devices are easily distinguishable in the time domain (Figure 3a). The slight asymmetry in the frequency response that corresponds to the transferred signal between the IDT, reflector, and IDT is typical for the YZ-cut of LN.

**Figure 4:**
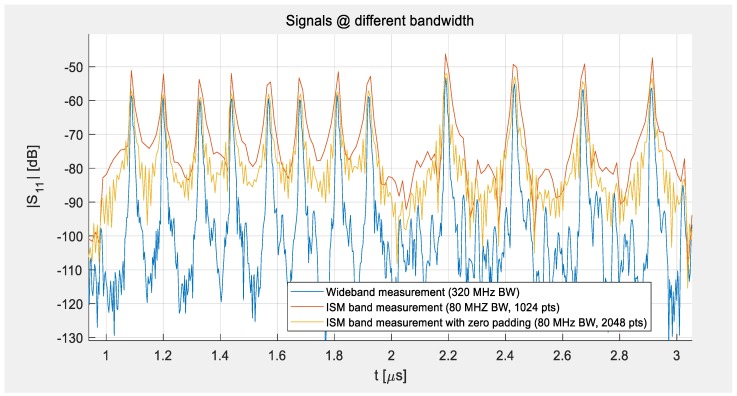
Peaks of the impulse response of the four-sensor-system as measured at different bandwidths at room temperature.

Figure 4 demonstrates how the bandwidth of the system influences the width of the sensor peaks in the time domain. The blue curve was measured at a wide bandwidth of 320 MHz and shows the full performance of the devices under laboratory conditions. It demonstrates very narrow peaks that can be easily separated from the signals of the other sensors and a good SNR. In comparison to the data shown in Figure 3a, all four sensors were here measured simultaneously. The red line shows the results if the bandwidth was limited to 80 MHz, allowing for free field measurements, and the number of points was reduced to 1024. This low number was applied to make the present S-parameter measurements comparable to the signals that a reader can deliver. While no restriction in measurement time was set for the VNA measurement, the reading system should be able to read, analyze, and transfer the data to the base station at least a few times per second. This limits the reasonable number of sampling points to about 1024. The yellow curve demonstrates that zero padding to 2048 points before IFFT allows the resolution to be improved via interpolation of the sampling points without increasing the time it takes for a measurement sweep, but only the processing time and the amount of memory needed in the reading system. This method was used during the range measurements described in Section 3.3 and is always applied during a readout-analysis with the reading unit.

### 3.2. Temperature Measurement

The sensors were installed inside a tube furnace at different distances to the heating coils to create different temperatures. The temperature was increased between 30 and 270 °C in 40 °C steps and held constant at five levels for ~4 h each. The measured S-parameters of all sensors were combined and processed by MatLab^®^ as described in Section 2.

**Figure 5:**
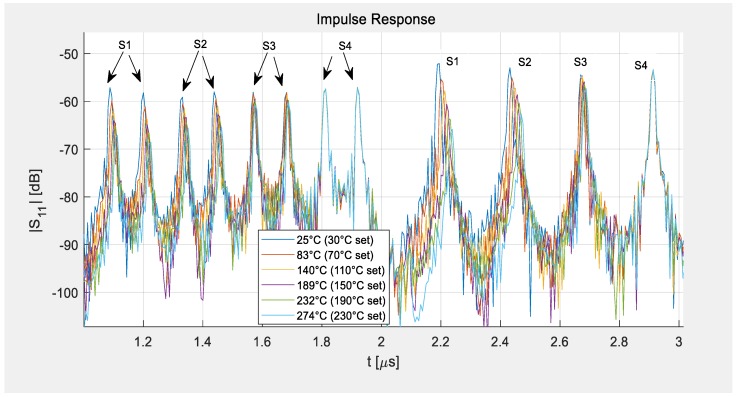
Shift of delay time due to heating of the sensors. Sensor 1 demonstrates the full shift of the delay time as it was placed at the center of the furnace, while the other sensors see less and less heat and sensor 4 is practically at room temperature and hence no time-shift occurs.

**Figure 6:**
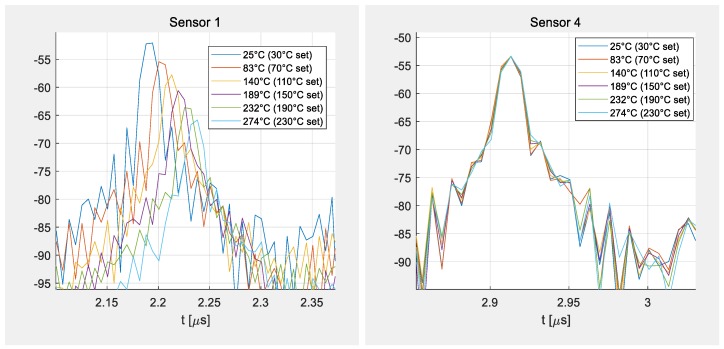
Details of Figure 5 showing the shift of the last peaks of sensor 1 (left) and sensor 4 (right)

Figure 5 and Figure 6 show selected data taken at different temperatures with a bandwidth of 80 MHz and zero padding of 2048 points. The figures demonstrate the shift of the delay time with the temperature. Sensor 1 was placed in the middle of the furnace, close to two reference sensors (K-type thermocouples), showing considerable time shifts for each temperature level (Figure 6a), whereas sensor 4 was kept outside of the heated area behind the heat shield and was hence hardly influenced by the heating so almost no time shift is visible (Figure 6b). Sensors 2 and 3 were placed in between sensors 1 and 4, showing the decreasing heat levels at their individual positions in less distinct time shifts. The temperatures given in the legend of the figures correspond to the temperatures set in the controller of the furnace (marked as set) and the data from the reference sensors and apply only for sensor 1. The other sensors show smaller time shifts as they were heated less.

**Figure 7:**
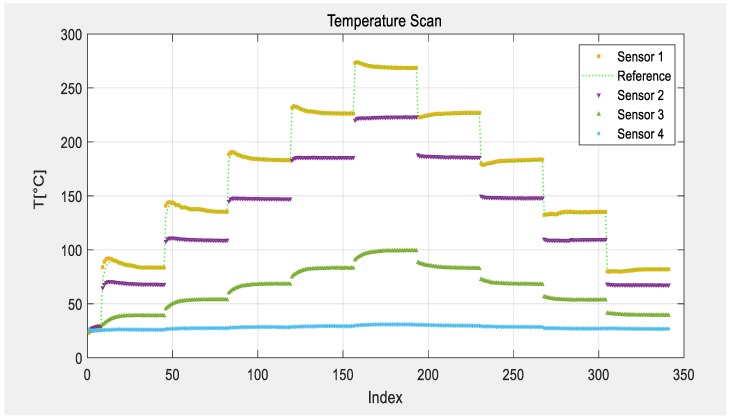
Temperature scans with four SAW sensors at different temperatures inside a furnace.

The temperature in the center of the furnace was set to 30, 70, 110, 150, 190, and 220 °C and the tube was evacuated to 1 mbar. Each plateau was set for 4 h to simulate a slow curing process in near thermal equilibrium of each level. The comparison with two reference sensors reveals that the temperature inside the furnace is higher than the programmed temperature level at the controller of the furnace. Especially, directly after a heating step, some overshooting of the furnace is visible from the two reference thermocouples. Figure 7 shows the data taken from the four SAW sensors after post processing in MatLab^®^. The TCD value in the data analysis was adjusted to fit to the reference sensor readings. This corresponds to a usual calibration step accounting for slight stresses on the chip due to thermal coefficient of expansion (TCE) mismatch between the package, glue, and chip, and heat transfer. One can see that sensor 1 follows the temperature curve of the reference quite well and that all four sensor readings could be analyzed despite their different temperature regimes and hence time shifts.

### 3.3. Wireless Reading

An extensive study was performed to determine the possible readout distance and resolution of the system when all constraints were applied: Band limits ~2.4–2.48 GHz; maximum power emitted by the transmitting antenna: 10 mW; and additional damping by cables.

These experiments demonstrate the main factors, which affect the system performance in wireless readout:

- Emitted power.
- Antenna gain and thus size of the reading antenna and sensor antenna.
- Quality of the reader system (sampling frequency and averaging).
  ○ Switched–frequency stepped continuous wave (S-FSCW).
  ○ Frequency modulated continuous wave (FMCW).
- Cables.

**Figure 8:**
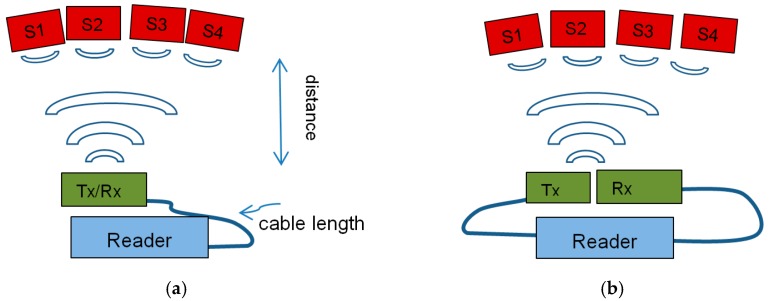
Symbolic drawing of the test setup for (**a**) single antenna and (**b**) two-antenna setup. Tx is the emitting antenna, Rx the receiving one.

In our measurement setup, four sensors of different designs, B 1–4, were each equipped with a slot antenna and placed within the cone of the same reading antenna. All sensors were read out by the same interrogation sweep. A metallic antenna was used on the sensor side for high temperature robustness, while commercially available antennas (PCB based) were used on the reader side assuming that they could be placed in a cooler area. Figure 8a depicts a schematic of the setup used for the data given in Table 2 and Table 3.

As reader units are relatively expensive RF systems, one additional goal was to check the possibility of accessing several measurement locations with one reader. The existing readers allow switching between four input channels, but such an extended configuration often requires the installation of longer cables between the reader and reader antennas to extend the distance between the measurements positions.

While our tests were all done with only one reading channel, we varied the cable length and assessed the achievable reading distance (between sensor and reader-antenna) and the resulting resolution to check the limits of such an approach.

The sensors were designed for operation in the ISM–band of 2.45 GHz, so the power for sensor applications was limited to 10 mW. This means the emitted power must be adjusted according to the gain of the emitting antenna not to exceed this limit. The experiments were performed with two different reader units, developed by CTR, an S-FSCW and an FMCW. A description of the reader principles can be found in [13].

S-FSCW and FMCW require different operation parameters for optimal reading (averaging, amplification). For both readers, various cable lengths and antennas were tested. To make the results comparable, the parameters were set in all configurations so that the emitted power considering the antenna gain and losses in the cables did not exceed 10 mW and the sampling frequency of the sensor readout was 5 Hz. All measurements were done using a 9 dBi slot antenna on the sensor side.

A two-antenna configuration on the reader side was also tested (Figure 8b). Here, a high gain (18 dBi) antenna was used as the receiving antenna (Rx) while the sending antenna (Tx) was kept at 9 dBi. Compared to a single 18 dBi TxRx setup, the reader output power can be kept higher and about 9 dBi are gained in the receiving part compared to a 9 dBi-antenna-setup.

**Figure 9:**
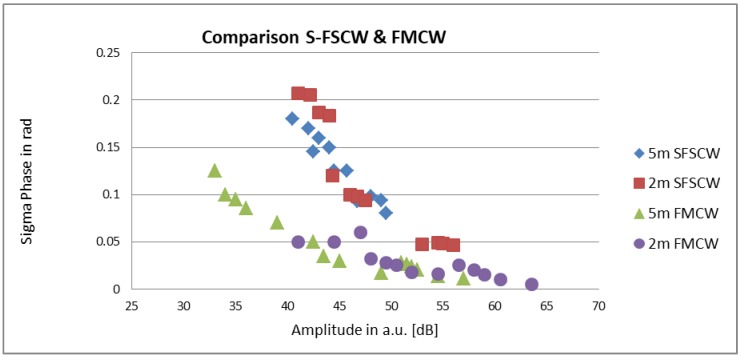
Comparison of the phase resolution of the S-FSCW and FMCW reader as a function of signal strength.

The phase resolution was measured for the two reader systems as a function of the signal amplitude. This data is shown in Figure 9. The given amplitude values are only for comparison and do not give absolute values as they comprise the amplifiers and ADCs in the reader system. The different signal strengths were induced by various reading distances and cable lengths. The experiments demonstrate clearly that the achievable resolution depends strongly on the signal amplitude. Because of the different reader architectures and the resulting readout strategies of the two reader types, the main difference between the system performances is mainly because a readout sweep of an S-FSCW unit takes more time than a sweep using a FMCW unit. To still be comparable, an equal sampling rate of 5 Hz was set up for both systems, resulting in no averaging for the S-FSCW reader in these experiments, whereas an averaging of 100 was applied for the FWCW. If a slower sampling rate is acceptable, the resolution of the system can be increased by applying higher averaging (see footnote in Table 2).

The measured phase and temperature resolution as a function of the antenna distance and the corresponding sensor peak amplitudes are shown in the following tables. The experiments were performed with an increasing cable length between the reader unit and reading antenna. The cables added losses of ~0.5 dB/m to the system. Different amplitude values occurred as longer delay times result in higher attenuation of the peaks. The criterion for the maximum readout distance between the reading antenna and sensor antenna was limited by a phase variance of 0.2 rad, which corresponds to a temperature resolution of ~0.15 °C. If less resolution is acceptable, a longer reading distance can be achieved.

Table 2 gives the results for the S-FSCW system. The largest reading distance can be reached with the shortest reader—antenna—cable. This is understandable as the cable losses in the receiving path cannot be compensated by a higher emitted power. The data in Table 3 for the FMCW reader shows a similar behavior, with, generally, better resolution at the same sampling frequency. For both systems, the reading distance was limited to around 1 m for the 9 dBi antenna or 50 cm for a cable length of 10 m. In all experiments, the variance of the phase reading was measured for all peaks. To simplify the display of the data, a range of amplitudes is indicated in the tables and only the highest value for the variance is shown. The shown temperature resolution was not measured directly but was calculated from the phase data.

Longer distances can be achieved with 18 dBi antennas especially if a two-antenna configuration is used (Figure 8b). The readers allow the transmitting and receiving path to be separated. If a smaller antenna with lower gain is used in the emitting path, the power can be adjusted to this antenna gain, while the receiving path benefits from the higher susceptibility of the 18 dBi antenna. With the two- antenna configuration, the losses within the 10 m of cable can be compensated to reach a reading distance of ~1 m, resulting in a phase resolution of 0.04 rad, or the reading distance can be increased to ~2.5 m when only a short cable is applied (<2 m), showing a phase resolution of 0.13 rad with the FMCW system.

## 4. Discussion

The goal of this work was to realize a multi sensor temperature readout based on SAW technology for the operation range from room temperature up to 300 °C and performance estimations for an attached antenna RF link.

Resonator based solutions were considered but not implemented, as the band limits of the ISM-band would restrict the applicable sensing temperature range too much. Instead, wide band reflective delay lines were designed and fabricated to compensate for the estimated frequency shift due to temperature changes and to move the signal evaluation towards the less restricted time domain. While the bandwidth limits of the ISM-band, which were observed during the measurements with the reader units, limits the system resolution in the time domain, the layout of the devices was chosen such that it ensured sufficient separation of the peaks even at big mutual temperature differences between the sensors. We demonstrated a successful simultaneous cable-bound readout of the sensors from RT to temperatures of ~270 °C, and the maximum temperature difference between two sensors was ~240 °C.

Wireless readout performed at room temperature shows that the devices can be interrogated to a distance of about 1 m if 9 dBi antennas are applied, the ISM- regulations for “non-specific short range devices” are observed (10 mW total radiation power at the transmitting antenna), and a harsh criterion of 0.15 °C is set for the temperature resolution. Provided that larger antennas can be used or if a lower resolution is acceptable, longer reading distances are possible. The easiest way to increase the readout distance is by simply raising the RF power. This is possible for applications within RF shielded closed areas or if special operation permissions are granted.

The current SAW sensor chips have a surface area ~6 mm × 1.25 mm. The delay path used for temperature measurements has a length of ~1.8 mm and the derived temperature corresponds to the mean value along this length. Hence, no real point like measurements can be performed.

The design allows only limited use in the presence of steep temperature gradients. The behavior and the limits for such situations where temperature gradients occur should be further investigated as well as the long-term temperature stability of the sensors as the sensor material (LN, LT) degrades when exposed to elevated temperatures, especially in chemically reactive atmospheres (e.g., in lab air, NOx, organic carbon based residuals). A possibly way to increase the temperature stability would be to apply a similar design to stoichiometric lithiumniobate [39].

Finally, this work demonstrated that four individual temperature sensors based on SAW reflective delay lines can be interrogated at once without interference by a single reader antenna while observing the restrictions of the ISM-band regulations.

## Figures and Tables

**Table 1:** Comparison of delay line and resonator SAW sensors.

Delay Lines	Resonators
Change of delay time is measured: material constant is temperature coefficient of delay (TCD)	Frequency shift is measured: material constant is temperature coefficient of frequency (TCF = −TCD)
Wide band device: sharp peaks in time-domain	High Q-factor needed for sharp peaks in frequency-domain -> narrow resonance.
Differential measurement is easy due to multi peak design	Differential measurement requires multiple resonators
Wide band operations allow for wide temperature shift	Too wide temperature shifts might shift the sensor’s resonance frequency out of the dedicated ISM-band
Devices are relatively long ~>4–6 mm, measurement corresponds to mean value over delay path ~2 mm	Resonators can be small and allow point-like measurements
Sophisticated phase tracking required	High resolution frequency reading needed

**Table 2:** Range measurements with S-FSCW reader, averaging = 1 and 9 dBi antennas.

Cable Length [m] ^1^	Distance [cm] ^2^	Amplitude [dB]	σ_phase_ [rad]	σ_T_ [°C]
2	50	50–56	<0.05	<0.04
	100	40–48	<0.12	<0.09
	120	38–44	<0.20	<0.14
5	50	45–50	<0.10	<0.07
	100	40–46	<0.15	<0.11
	110	35–43	<0.18	<0.13
10	50	38–40 ^3^	<0.12 ^3^	<0.08
	100	---	---	---

^1^ Between reader and reading antenna. ^2^ Between reading antenna and sensor antenna. ^3^ Averaging 10.

**Table 3:** Range measurements with FMCW reader, averaging = 100 and 9 dBi antennas.

Cable Length [m] ^1^	Distance [cm] ^2^	Amplitude [dB]	σ_phase_ [rad]	σ_T_ [°C]
2	50	58–62	<0.025	0.02
	100	42–48	<0.03	0.02
	140	40–45	<0.07	0.05
5	50	50–56	<0.02	0.01
	100	43–48	<0.05	0.04
	140	33–40	<0.13	0.09
10	50	30–40	<5	3.5
	100	---	---	---

^1^ Between reader and reading antenna. ^2^ Between reading antenna and sensor antenna.
